# Effects of the Educational Use of Music on 3- to 12-Year-Old Children’s Emotional Development: A Systematic Review

**DOI:** 10.3390/ijerph18073668

**Published:** 2021-04-01

**Authors:** José Salvador Blasco-Magraner, Gloria Bernabe-Valero, Pablo Marín-Liébana, Carmen Moret-Tatay

**Affiliations:** 1 Department of Teaching of Musical, Visual and Corporal Expression, Faculty of Teacher Training, Universitat de València, 46010 Valencia, Spain; j.salvador.blasco@uv.es (J.S.B.-M.); pablo.marin-liebana@uv.es (P.M.-L.); 2 Faculty of Psychology, Universidad Católica de Valencia “San Vicente Mártir”, 46001 Valencia, Spain; mariacarmen.moret@ucv.es

**Keywords:** emotional education, music education, school environment, systematic review

## Abstract

Interest in the study of emotions in education has grown in recent years. Some of our modern challenges, such as constantly adapting to new scenarios or the need for team work have justified the introduction of emotional competence into educational systems, while diverse studies confirm the relationship between music and emotional intelligence, so that the former could be used as a tool to develop the latter. The aim of this work was to examine the evidence for positive effects of music on the emotions of 3- to 12-year-old children, to which end a systematic review was carried out. Two reviewers independently evaluated 424 studies that were identified in MEDLINE, Psycinfo, and CINAHL databases, in order to determine whether they met the stated inclusion criteria. A total of 26 articles were selected for review. The results suggest several beneficial effects of music on children’s development, such as greater emotional intelligence, academic performance, and prosocial skills. It can therefore be concluded that music should be used in school settings, not only as an important subject in itself, but also as an educational tool within other subjects.

## 1. Introduction

The study of emotions is a subject that has raised researchers’ interest, being extended to fields ranging from philosophy, education, or psychology to health sciences. Regarding the field of education, a century ago John Dewey pointed out the importance of the social and emotional nature of the classroom and the relationship between social processes and learning [1]. However, the increase in the study of emotions in the area of education during the last two decades has given rise to a more humanistic, holistic, and socio-emotional approach to educational activity [2]. Salovey & Mayer [3] were the first to define emotional intelligence as “the ability to monitor one’s own or others’ feelings and emotions, to discriminate among them and to use this information and to guide one’s thinking and actions”. Since then, a large number of programs have emerged aiming to help educators prevent problem behaviours and to promote children’s health and character development [4]. In 1994, the Collaborative to Advance Social and Emotional Learning (CASEL) was created, an organisation whose aim was to promote and implement social and emotional learning as an integral part of teaching in schools. [5].

UNESCO´s well-known Report of the International Commission on Education for the 21st Century, entitled Learning: The Treasure Within [6], established four pillars on which the new education of the 21st century was based: learning to know, learning to do, learning to live together, and learning to be. The last two are closely related to emotional intelligence. Since then, research on the importance of emotional education in the field of education has not stopped growing in different scientific disciplines, especially in Psychology, Neurosciences and Behavioral Sciences [7]. Thus, for example, in the field of psychology there is a wide variety of works that address the study of emotions in the educational context from different topics, such as the importance of emotional competence in the classroom [8,9]; emotions and emotional regulation in the classroom [10,11]; or the importance of goals in the emotional experience of academic failure [12]. The field of neuroscience has produced numerous studies on the development of emotional regulation and the possible implications for education [13]; as well as the implications of affective and social neuroscience for educational theory [14,15,16]. Lastly, in the Behavioral Sciences, one can find studies as diverse as the essential characteristics of educational programs for students with emotional and behavioral disorders [17] or the professional preparation for teachers to effectively implement evidence-based practices for students with Emotional Disabilities [18].

The reasons for the significant increase in research on the relevance of emotional education in the specific educational contexts have been, in part, the fast and relentless global technological and economic and social changes which have created previously unimaginable pressures and challenges on the younger population, especially on children [19]. Moreover, the new challenges posed by today’s society demand future professionals who are able to learn constantly by working in a team [20], which is a challenge in interpersonal emotional management. It is therefore necessary to have eminently social individuals with a high capacity to adapt to the constant changes that today’s society demands [21]. For this reason, the educational systems of the most developed countries include in their educational programs the development of individual’s emotional competencies [22]. Social and emotional education is defined as “the educational process by which an individual develops intrapersonal and interpersonal competence and resilience skills in social, emotional and academic domains through curricular, embedded, relational and contextual approaches” [1] and all children should develop it in order to achieve full and integral personal formation.

What role does music education play in emotional development? In recent years, music education has gained special relevance as part of the curricula of compulsory education in most Western countries [23], both for its learning benefits in itself [24,25,26], as well as for its ability to promote the learning of other disciplines [27,28]. Music has a remarkable capacity to express, transmit, and evoke various emotions and affections in human beings [29,30], regardless of their nationality or culture [31]. 

In the last two decades the research on music and its ability to generate emotions in humans has been systematized [32,33,34,35,36,37,38]. Numerous studies affirm the ability of music to trigger physiological responses, such as changes in the heart rate, skin temperature, and conductance, respiration and hormone secretion [39,40,41,42,43]. Other studies claim that music aids emotional regulation [44,45,46,47] and some have shown that music stimulates the cognitive aspect [48,49,50,51]. Ultimately, music is described as multidimensional and researchers have categorised it by its arousal properties, emotional quality, and structural characteristics [52]. 

The link between music and emotion has contributed to the value of music as a discipline that can be implemented in formal education to develop students’ emotional competence [2,53]. One of the advantages of musical activities is that they mostly require collective participation, which requires cooperation and coordination on the part of the members of a society [54], making them useful tools for the advancement of socioemotional development. In addition, the social interactions required for music-making offer many opportunities for students to develop their abilities to evaluate their own feelings and at the same time try to relate constructively to the feelings of others [55]. According to Pellitteri [56] there are five ways in which music education and social-emotional learning are complementary: music can be used as an emotional stimulus; it can be an aesthetic experience; it can be used for relaxation and imagery; music-making is a form of self-expression; and music-making can be a form of group experience [57]. 

Music education thus has a strong impact on children and young people´s intellectual, social, and personal development and therefore on pupils´ psychological well-being [58,59]. To our knowledge, no systematic review has been carried out on how the educational use of music affects the emotional development of children between 3 and 12 years of age. In order to answer this question, a systematic review was carried out to obtain as many studies as possible that explore this developmental stage. The information obtained from all of the studies on this specific subject was thus synthesized and partial or biased conclusions were avoided by referring to the available documents or the authors’ subjective inclusion criteria. 

## 2. Materials and Methods 

To find out the effects of the educational use of music on 1–3-year-old children’s emotional development, a systematic review was carried out following the Preferred Reporting Items for Systematic Reviews and Meta-Analyses (PRISMA) statement guidelines. The question under study was: How does the use of music in the educational field affect the emotional development of children aged between 3 and 12 years old?

The articles were selected in different stages by two independent reviewers, who independently extracted data from articles that had been deemed eligible in the selection stage. Discrepancies at further stages were resolved by consensus with a third researcher, so that the process can be described in two main stages. First, the researchers read the titles and abstracts individually, and secondly, the full text to finally compare agreement. The inclusion and exclusion criteria were the same in both stages. In the case of a disagreement, a third reviewer was consulted. The articles rejected in the first or second stage for not meeting the inclusion criteria had the reason for their exclusion described in the results section. Data extraction was based on the recommendations of the “Cochrane Handbook for Systematic Reviews”, including the following information: (i) general information about the study (e.g., author’s citation, and country of origin); (ii) methodology (e.g., duration/follow-up of the study and design and type of music intervention); (iii) information related to the sample (e.g., selection method, sample size, age and sex distribution); (iv) information related to the outcome (e.g., effects in the emotion variable); and (v) additional information (e.g., statistical methods involved or size effects). 

Studies were identified in the MEDLINE, Psycinfo, and CINAHL Web of Science and EBSCO databases through EBSCO and WOS (ISI Web of Knowledge) to determine whether they met the stated inclusion criteria. General search terms with the controlled descriptors for each database were used, employing the Medical Subject Heading (MeSH) from MEDLINE terms, and the descriptors and terms published in the literature. As this is a relatively recent field of study there is not yet a general consensus on the definitions of several analysis variables and categories on the relations between music and emotional development, we opted to use generic key words, which opened up a large number of papers. The two main words were *music* and *school*, with the addition of *school* to reduce the search to the educational field. 

To connect these terms, we used the Boolean terms “AND” and “OR” to expand and restrict the search spectrum. In addition, a manual search was also performed. The total electronic search of all databases was performed between August and December 2020. The final syntax is defined as follows: “Music” AND “Emotion” AND “School”. In this way, we tried to restrict the sample to studies that focused on the relationship between music and emotions in educational contexts.

### 2.1. Inclusion Criteria

To be included in the review, articles had to meet the following requirements: (i) the sample of a study had to be children between 3 and 12 years of age, i.e., who were in pre-primary or primary school, to adjust the research question to the selected age range; (ii) it had to be an empirical study (i.e., cross-sectional, cohort, or case-control studies) to guarantee any conclusions drawn from our observations of reality; (iii) it had to measure the role of music in emotion so as to draw conclusions on the effect of music in primary education on the children’s emotional development; and (iv) it must have been published since 2000, in order to analyze studies from the last 20 years, when this new field of knowledge was developed.

### 2.2. Exclusion Criteria

Articles that met one of the following exclusion criteria were not added to the analyzed sample: (i) those not expressly measuring emotions, dealing with the subject matter in an indirect or secondary way, i.e., those that did not directly deal with the relation between music and emotional development in spite of containing the key words; (ii) those that were non-empirical theoretical or bibliographical studies; (iii) those that were single case studies, due to the difficulty of generalizing any results obtained; (iii) those that included sample ages outside the selected range, i.e., studies on children less than 3 years old, adolescents and adults; and (iv) those that were grey literature or non-peer reviewed journal articles, to guarantee the quality of the reviewed papers.

## 3. Results

**Figure 1:**
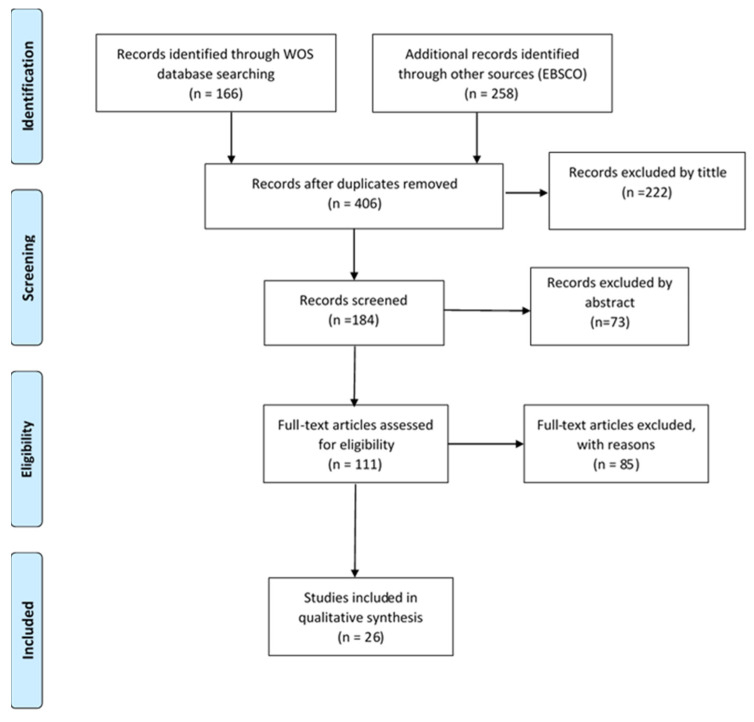
Preferred Reporting Items for Systematic Reviews and Meta-Analyses (PRISMA) flowchart to show study selection process [60].

The results obtained in the systematic review are presented below. After a selective process using the PRISMA protocol flowchart as a reference [60], the results of the systematic review are depicted in Figure 1. The sample analyzed reached a total of 26 scientific articles. The number of articles excluded according to the different criteria is shown in Table 1 and a summary of the content developed from the analyzed articles can be consulted in Table 2. Below is a description of the sample used and a narrative summary of the different papers, grouped according to subject matter.

### 3.1. Sample Description

**Figure 2:**
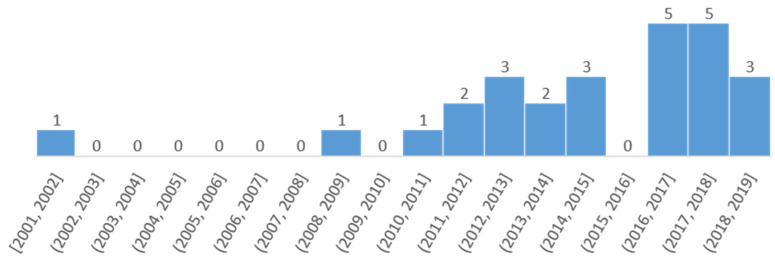
Evolution of publications.

Of all the articles analyzed, a total of 1954 subjects were selected (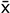 = 75.15, SD = 62.78). Publication dates were relatively recent with a range from 2001 to 2019 (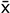 = 2014.96, SD = 3.96), experiencing linear growth since 2008 (see Figure 2). As for the educational context, 11.54% of the work was carried out in the field of early childhood education (3–6 years), 73.08% in primary education (6–12 years), and 15.38% in extracurricular activities (7–10 years). The topics identified were: the development of emotional intelligence (50.00%), divided into perception, assessment, and expression (42.31%), and emotional regulation (7.69%); the educational and formative benefits (42.31%); and the socioemotional benefits (26.92%), as shown in Table 3. However, no articles were found to address the levels of emotional facilitation and understanding identified by Mayer and Salovey’s model [87].

### 3.2. Combined Results

After analyzing the selected studies the results were organised into two lines: (1) dependent variables, a description of the socio-emotional effects/benefits of music; and (2) independent variables, different types of musical experience and their different effects. It should be noted that many authors did not make this distinction, while other co-relational studies did not follow a specific direction in the associations found. However, we attempted to structure these by assigning dependent and independent variables according to the interpretation of the aim of the studies.

### 3.3. Organization of the Information on Dependent Variables: What Emotional Effects Are Provided by Music?

This section was structured on the groups included in Table 3, in which the results are given according to: emotional intelligence, and the educational, training and socio-emotional benefits.

#### 3.3.1. Emotional Intelligence

After analyzing the selected articles we found 11 papers divided into two sub-topics: (1) emotional perception, appraisal, and expression, (2) and emotional regulation.

##### Perception, Assessment and Expression

Six studies addressed the role of music in relation to emotional perception and assessment. For example, Nieminen [65] observed that students in the first two years of primary school have the ability to identify greater happiness in pieces composed in the major mode than in the minor mode. This relationship was stronger in those with musical training, especially among the younger ones. In a similar vein, Schellenberg and Mankarious [68] measured perceptual differences between a group of students with and without musical training, finding that the former scored higher in identifying emotions in images and/or texts. However, the authors point out that this relationship appears to be mediated by IQ, which may be biased both in terms of participating in formal music education activities and identifying emotions on the basis of a measurement instrument based on visual and linguistic processes. 

In relation to specific interventions, Kim and Kim [73] found that the use of a music education program based on group instrumental performance improved students’ ability to recognise emotions. Katagiri [79] found in a study conducted with a group of children with autism spectrum disorder that teaching them to recognise emotion is more effective with music than when only verbal instructions are used. In addition, it was found that the use of background music associated with the emotions being worked on obtained better results than nursery rhymes, especially for the emotion of anger, as opposed to happiness, sadness, and fear. Of these above four studies it was deduced that musical entertainment and/or music education can help to recognise emotions in pieces of music, texts, and images, unlike other types of non-musical activities, and that this result can also be applied to specific populations such as children with autism spectrum disorder.

However, another study did not find any effect of music training on Emotional Comprehension in children who began the program with high levels of social skills [74], although it did provide a significant improvement in the children who had poor social skills at the beginning of the study. Along the same lines, Habibi et al. [64] compared the differences between children who participated in after-school activities in music, sports, or who had not enrolled in any specific activity. The study found no significant differences between the three groups in recognising emotional states by viewing pictures of eyes and empathising with the emotions of others, supporting the idea that the differences found in other studies are due to musical experiences.

However, the results in which no difference was found between the musically trained group and the other two groups could have been due to the short training time (5 days per week for two/three weeks). As the authors themselves propose, the differences associated with musical training were found in another longitudinal study with a 14-month training period with 5–7 year old children [88]. However, Habibi et al. [64] considered that their results also give some indirect support for the idea that the kinds of social and emotional skills reported in children who have studied music may be a by-product of music training and designed their study controlling the base line of previous skills in all of the groups, which provided a good base for a complete longitudinal study for the subsequent identification of whether the development of different skills in musical and non-musical adults is specifically due to musical training or to previous skills.

Three papers that deal with emotional expression agree that music can favor emotional expression. For example, Boone and Cunningham [62] found that, between the ages of 4 and 5 years, children are able to physically express some emotions that they perceive through music, such as happiness, sadness, anger, and fear (especially the first two). A study conducted with students from low socio-economic backgrounds concluded that using an Orff-based approach improved their ability to express emotions [78]. Improvement was also observed in a study in which a music therapy program was implemented [61].

##### Emotional Regulation

Two of the articles analyzed dealt with emotional regulation in relation to educational programs including music therapy and concluded that they favored emotional regulation. On the one hand, Brown and Sax [63] compared the emotional state and regulatory capacity of a group of students after attending a traditional education program or a program with a greater emphasis on arts education, including music education. It was found that individuals who participated in the latter showed a greater capacity to regulate emotions, both positive and negative. Moore and Hanson-Abromeit [61] observed that a music therapy program improved a range of behaviors associated with emotional regulation such as aggression, attention and both internal and external attitudes.

#### 3.3.2. Educational and Training Benefits

Eleven of the articles analyzed studied the effect of the use of music on the performance of school tasks. For example, Tricard et al. [77] found that video clips and background music to induce joy and sadness caused students who experienced a positive mood to score higher on deductive reasoning activities. Teske et al. [75] also found that students’ creativity was enhanced. On the other hand, Venegas et al. [76] found that the use of an interdisciplinary application that used music to support the learning of graphical representation in mathematics generated positive emotional levels in students. Similarly, another study found that greater use of music education increased motivation levels among students [63]. Rauduvaite [70] found that the introduction of urban popular music into the classroom can promote meaningful values in education, partly due to the emotional bond that students have with this type of repertoire.

However, music does not always have only beneficial effects. Su et al. [86] concluded that listening to background music while reading reduces students´ anxiety and improves some aspects of their reading comprehension, such as extracting explicit information and making direct inferences. However, other aspects with a higher level of interpretation, such as the integration of ideas or the evaluation of content, language, and textual elements, scored lower. Similarly, Rauduvaite [70] found that the introduction of urban popular music into the classroom can promote meaningful values in education, partly due to the emotional bond that students have with this type of repertoire. Soulier et al. [72] investigated the relationship between a music-induced mood and performance on spelling tasks, concluding that inducing negative emotions through sad music resulted in poorer performance. 

Other studies propose indirect methods to promote an emotional state that can improve the performance of school tasks. In the context of a remedial class, Pimenta and Trevisan [69] concluded that the introduction of music, especially choral singing, led to an improvement in the feelings experienced when attending these sessions and a new way of relating to the class, as well as a greater interest and involvement in the class. In a similar way, Ramdane et al. [66] studied the beliefs of a group of Islamic education teachers about the effect of using singing in their classes, and found that the vast majority believed that singing gave them greater emotional awareness and motivation, which could lead to better learning. Similarly, another study found that the use of music with a strong religious-emotional component led students to improve their concentration and creativity [82]. 

Improving emotional skills can improve the results of music practice. A research study addressed the relationship between emotional skills and instrumental piano practice [80], obtained several results. Firstly, they found that identifying emotions helped students to integrate emotional expression into their piano playing and to play more fluently. Secondly, the type of activities most effective in addressing emotional competence were improvisation and associating the pieces with personal experiences. Thirdly, emotional control allowed for greater organisation of the study, minimising moments of impatience, of playing fast and skipping steps, of not being aware of the music, and of not facing difficulties. Finally, bearing in mind that a public performance is conditioned by concerns about others’ judgements, as well as one’s own level of self-esteem, it was claimed that the variable under study could be enhanced by sharing feelings, collaborating, and helping peers, or achieving a goal through effort.

To sum up, the diverse papers analyzed provide empirical evidence of the effect of music in different areas of education, including the negative effects to be avoided (such as its interference with reading tasks that require a high degree of interpretation) and the positive ones that need reinforcement (e.g., a positive mood to promote learning).

#### 3.3.3. Socio-Emotional Benefits

Nine studies found that the use of music enhanced some socio-emotional benefits in diverse areas such as social skills, empathy, and reducing emotional problems. For example, Schellenberg et al. [74] found that students with a musical background scored higher in sympathy and prosocial skills, although only those with lower levels of musical performance. Another study concluded that singing music with a strong emotional component led students to improve in attitude and social skills such as teamwork [82]. Similarly, an intervention based on music education resulted in students developing pro-social emotions in relation to their peers with autism spectrum disorder, especially in cases where the latter were bullied [81]. Kawakami and Katahira [71] studied the relationship between empathic traits and liking sad music, concluding that those individuals with a higher level of concern for the negative experiences of others, greater capacity to adopt the perspective of the other, and greater development of fantastic imagination, experienced more positive emotions when listening to sad music. Porta [81] showed that film music makes sense of the audiovisual narrative and helps to hold children’s interest even when they lose the visual part. 

Regarding the approach to music education, Jeremić et al. [67] measured differences in the social-emotional competencies of children who were taught singing by a specialist teacher who used an active methodology in which she performed the songs, or by a non-specialist teacher who played the recordings on audio devices. Significant improvements in social-emotional competencies were found in the experimental group that had used an active method. For example, they were more empathetic towards those who had difficulty with intonation and felt more positive about singing individually. These results suggest that not all of the ways of using music obtain the same effects.

Other studies found a decrease in negative social-emotional attitudes when participating in certain intervention programs that included music. Thus, Ho et al. [83] studied the effect of an experience combining participation in a drumming group with educational counselling on students with low socio-economic backgrounds. The group made significant improvements in behaviors such as anxiety, depression, post-traumatic stress disorder, inattention, and some defiant attitudes. In the same direction, Kang [71] investigated the effect of a therapy based on the use of music (singing, listening, and performing) and sand play in children who had witnessed domestic violence. The author observed that individuals showed improvements related to emotional behavioral problems such as depression, anxiety, aggression, oppositional behavior, and post-traumatic stress. Finally, it was observed that the use of a music education program based on group instrumental performance reduced physical and verbal aggression [73].

Although there have not been a great number of studies performed that show the socio-emotional benefits, they all provide diverse methods for using music to improve these skills.

### 3.4. Organizing Information by the Independent Variables: What Types of Musical Experience Have What Types of Socio-Emotional Effects?

Ten studies used listening to music as the independent variable to compare its effects in different emotional areas and in general found them to be beneficial. Nieminen et al. [65] showed that children better identified their emotions according to major or minor chords. Su et al. [86] concluded that background music improved students’ moods, and thus indirectly other skills such as reading comprtehension. Porta [84] found sound held children’s interest even in the absence of images and that music had a significance for children in aspects related to emotions. Tricard et al. [77] found that videoclips and background music promote positive emotions in children and a significant improvement in deductive reasoning. Teske et al. [75] found that happy music promotes a positive mood and creativity. Katagiri [79] found that background music increases emotional comprehension in autistic children. Rauduvaite [70] found that popular music helped children’s education due to their emotional tie with this repertory. Finally, Boone & Cunningham [62] found that children could represent the emotional significance of music by expressive movements, especially the sad and happy moments. One of the controversial aspects was the effect of sad music on children: while Kawakami & Katahira [85] found that empathic children enjoy sad music, Soulier [72] found that inducing negative emotions through sad music reduced their concentration on spelling tasks. 

Fourteen studies used musical training as the independent variable and obtained evidence that it improved diverse socio-emotional competencies, including: identifying emotions in images and/or texts [68]; a greater capacity to regulate emotions [63]; concentration and creativity [82]; students’ ability to recognise emotions [73]; improved learning and mathematics [76]; a positive effect on children’s self-expression, self-efficacy, and social skills [78]; improved the feelings experienced when attending class [69]; greater emotional awareness and motivation from singing [66]; increased empathy and positive feelings for learning [67]; influenced the effects of emotional understanding [64]; influenced emotional awareness, regulation and autonomy [80]; increased pro-social behavior in neurotypical children in relation to the social exclusion of autistic children [74]; promoted the development of pro-social skills [81]; improved behaviors such as anxiety, depression, post-traumatic stress disorder, inattention and some defiant attitudes [83]; and increased children’s motivation and thus their learning capacity [66].

Finally, two studies used a musical therapy program as the independent variable and found improved emotional behavioral problems, including depression [71] and children’s emotional comprehension and emotional regulation [61].

## 4. Discussion

This systematic review seeks to understand the role that music plays in the emotional education of children in infant and primary education. The scientific literature indicates, on the one hand, that in all educational processes there is an emotional component that conditions the teaching-learning process [1]. On the other hand, today’s society demands individuals who are increasingly able to adapt to change [21], and with the skills to work collaboratively [20]. This has led to the implementation of various educational programs that seek to introduce the development of emotional competence in the classroom [4,5,22]. At the same time, some studies belonging to the fields of Psychology [8,9,10,11], Neuroscience [13,14,15,16], and Behavioral Sciences [17,18] have investigated the importance of emotional education in the education system. In addition, the central role of music has been demonstrated in aspects such as emotional expression, emotional induction, and emotional regulation [29,30,31,32,33,34,35,36,37,38,44,45,46,47].

Starting from this conceptual framework, this review aims to systematise the knowledge accumulated through the research works of the last two decades with samples made up of individuals from 3 to 12 years of age. The 26 articles analyzed indicate that publications on this topic have experienced a linear growth since 2008, that most of the research is carried out in the context of primary education, and that the topics most frequently dealt with are the development of emotional intelligence [87], educational and training benefits, and socio-emotional benefits.

With regard to the first aims, two studies have observed that students with musical training have a greater facility for recognising emotions [65,68]. The attribution of this effect to musical experiences is reinforced by a study that found no differences prior to such training [64,74]. Other studies have shown how certain educational interventions using music led to improvements in the emotional recognition of students [73,79]. However, caution should be exercised with these results, as Schellenberg et al. [74] found no differences between children with and without musical training, whereas Schellenberg and Mankarious [68] point out that the relationship could be mediated by IQ, which would act as a bias. In this regard, it would be interesting for future research to experiment with measurement instruments that do not depend on the cognitive abilities of the participants.

In relation to the two studies [64,74] that found no differences between the groups with and without musical training in emotional recognition skills, we consider that the results, in spite of being the opposite of those expected, do not invalidate the general statement that music favors emotional perception and assessment. This is because firstly it provides evidence that music is an especially efficient instrument for children who had poor social skills at the beginning of the study, and secondly it emphasises the need to control the participants’ basic levels and carry out longitudinal studies long enough to capture the improvements produced while establishing relationships that specifically assolciate these benefits with musical training. 

Similarly, it has been found that the use of proposals that include music improves children’s capacity for emotional expression [61,78], who are able to express perceived emotions through music from the age of 4–5 years old [62]. Emotional regulation also benefits when methodologies in which music plays a central role are used [61,63]. Therefore, the results obtained suggest that the use of music, whether in the form of specific ongoing training or one-off interventions, improves some aspects of emotional intelligence [87], especially emotional perception, expression, and regulation.

This is consistent with some studies that argue that music has a remarkable capacity to express, transmit, and evoke diverse emotions and affections in human beings. Thus, for example, Flores-Gutierrez & Diaz [29] claim that there are musical sequences that stimulate a specific group of emotions and others that evoke a more general response to a type of emotion that has a certain polarity, such as being pleasant and vigorous. Thus, in their study they find music that globally stimulates one of the four major axes defined in their circular affective model, such as pleasant (Mozart), unpleasant (Mussorgski), exciting (Metallica), or relaxing (Japanese music) emotions. For their part, Thompson & Quinto [30] claim that the power of music to elicit emotion lies in its ability to engage participants in tightly controlled synchronisation at multiple levels of abstraction. Music optimally recruits synchronisation processes that are ubiquitous in human behavior and that greatly influence our emotional lives.

This ability to express, transmit, and evoke diverse emotions and affects is, moreover, independent of the culture to which one belongs. Balkwill & Thompson [31] demonstrated that naïve listeners from different cultures obtained as high a level of agreement in their emotional responses to music as expert listeners, who were deeply familiar with the culture-specific cues embedded in the music samples. 

On the other hand, music helps emotional regulation. In this regard, Randall et al. [45] conducted research in which they found that using music to regulate a recently experienced emotion achieved the greatest hedonic success. For these authors, personal music listening is used as an independent regulatory resource, allowing listeners to achieve specific emotional goals. Saarikallio & Erkkilä, [47] conducted a study to recognise music´s role in mood regulation in adolescents. The study showed that listening or performing music has an impressive capacity to promote emotional self-regulation. In this regard, music offered adolescents resources to enhance and restore well-being, making their emotional lives more varied and colorful. In addition, Saarikallio [46] asserts that the general nature of music-related emotional self-regulation remains relatively similar throughout adulthood.

It therefore seems reasonable to think that the educational use of music may contribute to students’ development of some dimensions of emotional intelligence. However, none of the work addresses the levels of emotional facilitation and understanding reported by Mayer and Salovey [87] in their model. This could be due to the fact that these are higher level skills with a higher level of complexity and abstraction that are beyond the developmental possibilities of infant and primary school students. Future work should explore the possibilities of developing these levels of emotional competence in school-age children. 

In relation to the educational and formative benefits, the results indicate that music can play a role in triggering positive emotional states [75,76,77] and higher levels of motivation, concentration, and interest [63,66,69,70,82], which promotes learning in the classroom. It can also reduce the occurrence of negative emotional states such as anxiety and depression [86]. These benefits have been observed in relation to different school contents, such as improving deductive reasoning [77], creativity [75], graphic representation [76], reading [86], education in values [70], orthography [72], or instrumental practice [80].

These results are consistent with other research that has found music activities such as instrumental performance or improvisation stimulate cognitive processes [48,49,50,51], and that they are able to support learning in other disciplines. Johnson & Memmott [27], in a study of 4739 children, demonstrated the relationship between the quality of music instruction and academic performance on standardised tests in English and mathematics. For these authors, music is a very useful tool to support academic performance. On the other hand, Rickard et al. [28] conducted a study with 151 schoolchildren on the impact of school music programs on verbal and visual memory processes over a two-year period. The results revealed that schools with high-quality music programs performed better on standardized tests than students in schools with lower-quality music offerings.

Finally, in terms of socio-emotional benefits, the articles reviewed agree that both music training and music education can be beneficial for students’ social and emotional development [74], such as the fact that the use of music in educational and therapeutic processes [81,82] can have a positive impact on the development of social skills in which some kind of emotion comes into play, such as empathy, teamwork, the development of a pro-social attitude or self-esteem. Similarly, they can contribute to the reduction of negative socio-emotional attitudes such as depression, anxiety, aggression, inattention, defiant and oppositional behavior, or post-traumatic stress [71,73,83]. These benefits coincide with some of the challenges posed by today’s society, which requires social subjects with high intrapersonal and interpersonal skills that allow them to work in teams and adapt to change [1,20,21].

Specific educational programs that encourage the development of pro-social skills include participation in a drumming group [83], group musical performance [73], or training in singing through a specialist teacher who used her voice in the classroom [67]. These results seem to indicate that the use of music has the capacity to bring social-emotional benefits insofar as it is experiential and fosters personal relationships. This is consistent with some authors who point out that music often involves a series of social interactions that favor socio-emotional learning [55,56,57]. Moreover, it coincides with the ethnomusicological perspective that conceives of music as a socio-cultural phenomenon that requires cooperation and coordination [54]. This perspective would also contribute to broadening the theoretical basis that justifies the use of active methodologies in the classroom as opposed to more traditional models focused on theory, technique, and individual learning [89,90,91,92]. In further research, it would be useful to study not only the greater or lesser use of music, but also the methodology implemented. In this regard, the comparison between the effects produced by traditional, active, and critical pedagogical models would be a valuable contribution to the field of emotional education, as they could be used to guide teachers in their educational practices and to design both initial and ongoing professional training programs.

Regarding the different musical experiences, it was found that both exposure to music, musical entertainment, and musical therapy programs provide improvements in the emotional sphere.

## 5. Conclusions

This systematic review found that using music in the education of 3–12 year olds can have a positive effect on their emotional development. We first found that it can contribute to the development of emotional intelligence, especially with regard to emotional perception, expression, and regulation. The students that took part in activities using music were more capable of recognising and expressing their emotions and regulating their emotional states, such as aggression or anger. Secondly we found that music is capable of providing educational, formative, and socio-emotional benefits. In this regard its emotional impact can improve aspects such as deductive reasoning, creativity, graphic representation, reading, spelling, education in values, or practical instrumental skills. It can also boost attitudes such as sympathy, empathy, and other prosocial skills, and reduce anxiety, depression, and defiant attitudes. It is therefore recommended that music be incorporated into the different curricular levels, educational contexts, and areas of knowledge. Likewise, the establishment of educational policies aimed at guaranteeing universal access to musical training is suggested, as well as an increase in the presence and recognition of this discipline in formal educational contexts.

Most of the limitations encountered in this study were due to the considerable heterogeneity in the different studies selected, which made it difficult to integrate them into a single framework. All the studies used different independent variables, such as musical listening, entertainment, or music therapy, and different dependent variables such as different emotional effects or in the development of socio-emotional skills. We therefore had to integrate and synthesize the information into a scheme to make sense of the results. However, our double approach to structuring the results (first by the socio-emotional effects and then by the musical variables) allowed us to better structure them, although it was a difficult and complex task. 

We should first of all point out that the studies came from different disciplines such as musical education, music psychology, music teaching, music therapy, etc., and thus different researchers used their own theories, methods, nomenclatures, and instruments, which made it difficult to combine the results in a common language. The second difficulty was the lack of a classification with a wide consensus of positive emotions or emotional skills. Psychology is known to classically study psychopathology and has classified the universally accepted diverse psychological disorders (e.g., Diagnostic and Statistical Manual of Mental Disorders (DSM-V) [93] and the International Classification of Diseases (CIE10) [94]). These serve as a guide to researchers who can compare their results using these classifications. However, until the emergence of Positive Psychology in the first decade of the 21st century this discipline had not focused on the positive aspects of human existence, so that there were still no official classifications of the positive emotional sphere that were generally accepted by all researchers 

The present work aimed to find positive emotional aspects, and although we used studies from the year 2000 these were not based on a common taxonomy regarding the measurement and classification of these effects. However, we had serious difficulty in integrating the results into a common scheme, although we got some support from Mayer & Salovey’s Theory of Emotional Intelligence [87], which includes different emotional facets and skills and is widely recognized in scientific fields. Thirdly, we should point out that there are overlaps and covariances among the dependent variables (e.g., if we improve emotional recognition the socio-emotional skills will probably also improve) and in the independent variables (e.g., musical entertainment programs include listening and music therapy). This makes it difficult to ascertain the relations between the variables and alerts us to the need for a more experimental approach in this field that uses common categories to construct more solid knowledge. 

Fourthly, we were not able to extract solid syntheses and conclusions from homogenous groups in diverse variables of interest (e.g., specific ages, music styles, different levels of empirical evidence) due to the lack of appropriate studies. We opted for a generic search in order to deal with the wide range of papers. A narrower search would have provided greater precision with more homogeneous results but less information, counting on a small number of papers for the systematic review. We therefore consider that our approach achieved a balance between information and precision, since it allowed us to draw a map of the present situation and integrate this information into a joint scheme. 

The wide fragmentation of the papers is an indication of the relatively recent emergence of this field of study, which associates music education with its emotional effects. However, we consider that studies such as this will allow us to detect the obstacles and deficiencies in order to go on constructing the basis of a common language to improve the comparison of the results and the advances in clarifying the empirical evidence in relation to the effects of musical education in the socio-emotional area. 

## Figures and Tables

**Table 1:** Table showing reasons items were excluded.

Exclusion Criteria	Total Items Excluded
The work does not specifically address the research question	259
Not an empirical study	58
Children were not within the age range 3–12 years old	41
Sample of participants included mixed ages	12
Sample *n* = 1	3
Not an article	7

**Table:** (**A**)

Study	N	Age	Educational Setting	Primary Purpose of Study	Study Design	Music Variables	Emotion Variables	Music Style	Relevant Data Collection Measures
Moore & Hanson-Abromeit [61]	8	3 to 5	Preschool	To examine feasibility and preliminary efficacy of the Musical Contour Regulation Facilitation (MCRF) intervention, a multi-session strategy for promoting ER development in preschoolers	Case study	To participate in the MCRF intervention	Emotion understanding accuracy; emotional regulation; child’s expression of emotion	Not specified	Language Development Survey (part of ASEBA; Achenbach & Rescorla, 2000); Emotion Regulation Checklist (ERC; Shields & CIcchetti, 1997, 1998); Child Behavior Checklist (CBCL) and Caregiver-Teacher Report Form (C-TRF), both part of the ASEBA (Asenbach & Rescorla, 2000)
Boone & Cunningham [62]	47	3 to 6	Preschool	To determine if children can accurately express the emotional meaning in music through expressive movement	Observational	12 music segments previously identified as belonging to one emotional category (happiness, sadness, ange or fear)	Happiness, sadness, anger, or fear	(1) Romanian Rhapsody, Opus 11/Enesco; (2) Peter Gynt; Ase’s Death, Suite No. 1, Opus 46/Grieg; (3) Theme to Lifeforce/Mancini; (4) Surprise Attack The Wrath of Khan/Horner; (5) Concerto in D, Opus 35/Tschaikovsky; (6) The Humorous Song/Lyadov (7) The Rite of Spring/Stravinsky (8) The Red Poppy, the Russian Sailor’s Dance/Gliere (9) Winter Games/Foster (10) Anvil of Crom, Conan/Poledouris (11) Venus/Holst; (12) The Walls Converge, Star Wars. Williams	Ad hoc observational registration
Brown & Sax [63]	205	4	Preschool	To examine the impact of arts-integrated preschool programming on the emotional func-tioning of low-income children at risk for school problems	Correlational	Participate in an arts integrated preschool program	Emotion understanding, empathy, emotional lability, anger reactivity, negative emotion intensity	Not specified: music, dance, and visual arts class	Peabody Picture Vocabulary Test-III (PPVT-III; Dunn & Dunn, 1997); Emotion Regulation Checklist (ERC; Shields & Cicchetti, 1997); and an adapted version of the Affex system, which is grounded in DET (izard, Dougherty, & Hembree, 1989)
Habibi et al. [64]	45	6 to 7	Out of school	To determine whether children who participate in musical training were different, prior to training, from children in the control groups in terms of cognitive, motor, musical, emotional, and social measures	Cross-sectional between subjects; experimental (controlled trial, non-randomized sample)	To be involved in a systematic and high intensity musicaltraining, and music perception	Emotional recognition and empathy	Not specified. Gordon’s primary measures of music audiation (PMMA) requires children to listen to a recording of 40 pairs of simple rhythms and 40 pairs of tone sequences and make a same/different judgment for each pair by circling a pair of same or different faces (Gordon, 1986).	Auditory analysis test (Rosner and Simon, 1971); Gordon’s primary measures of music audiation (PMMA; Gordon, 1986); Reading the Mind in the Eyes (Baron-Cohen et al., 2001); Index of Empathy for Children (Bryant, 1982); video emotion test (Goldstein adn Winner, 2012); Helping and sharing test (ad hoc)
Nieminen et al. [65]	127	6 to 9	Elementary school	To investigate the effects of age, gender, and music education on musical preference, musical emotion recognition, and the use of the aesthetic categories for music	Cross-sectional between subjects; experimental (non-randomized sample)	Musical preference and musical mode (major, minor and free tonal)	Beauty, ugliness, happiness and sadness	Three short musical pieces (20 s each) resembling familiar children’s songs of Western music	Ad hoc questionnaire and task
Ramdane et al. [66]	186	6 to 12	Elementary school	To investigate the usefulness of using music and songs by Islamic Education teachers	Correlational	Using music at the classroom	Emotional awareness	Not specified	Ad hoc questionnaire and interviews
Jeremic et al. [67]	89	7	Elementary school	To study the effect of vocal performance as a teaching method in relation to the social-emotional competencies (SEC) of pupils	Cross-sectional between subjects; experimental (controlled trial, non-randomized sample)	The vocal performance teaching method	Empathy, impulsivity, emotional control and reactions	Songs by ear where the basic psychological processes take place when singing accompanied by elements of vocal technique. Perceptual characteristics include singing by ear, tone of voice and resonance	Scale for assessment of social-emotional competencies of students (SEC)
Schellenberg & Mankarious [68]	60	7 to 8	Out of school	To examine whether music training in childhood is predictive of understanding emotions	Correlational	Child’s history of music lessons	Emotion recognition	Not specified	Test of Emotion Comprehension (TEC; Pons & Harris, 2000), and Weschler Abbreviated Scale of Intelligence (WASI; Weschler, 1999)
Pimenta & Trevisan [69]	31	7 to 9	Elementary school	To analyze the influence of music in promoting changes in the relationship established by students of recovery class with school activities	Case study	Activities with music, musical compositions	Aggressive, loud and disinterested in the classroom versus calm, participatory and interested in music gatherings	Not specified	Ad hoc instruments
Rauduvaite [70]	70	7 to 9	Elementary school	To analize how popular music can educate	Observational	Popular music repertoire	Positive emotions towards learning	Popular music. “Mom” song (music by R. Sileika, lyrics by D. Teiserskyte). “You and Me” song (music by V. Noreikis, lyrics by A. Cibarauskas) Listening to “The Return of Spring” by D. and G. Gibsons	Ad hoc instruments
Kang [71]	3	7 to 10	Elementary school	To examine the possibility of supportive music and imagery (MI) in addition to sandplay as a therapeutic treatment to improve emotional and behavioral adaptability for child witnesses of domestic violence	Case study	Create music through improvisation with musical instruments or their voices	Emotional behavioral problems including depression, aggression, anxiety, oppositional problem, PTSD, peace	Hello song, Goodbye song. L’Arlesienne Suite No.1 Adagietto F major by Georges Bizet. A Korean popular song, “A Firefly,” whose lyrics are about parting and loneliness. Music for the children and the musical instruments in the therapy room while listening to the music.	Korean Child Behavior Checklist (K-CBCL)
Soulier [72]	234	7 to 11	Elementary school	To study the effect of an emotional induction by music on the grammatical spelling performances of primary-school pupils	Cross-sectional between subjects: experimental (non-randomized)	Exposure to musical excerpts judged to be emotionally neutral, happy, and sad	Positive, negative emotions and neutral emotions	Musical excerpts with emotional valences: positive, negative and neutral: (1) Adagio en sol mineur—Tomaso Albinoni; (2) Nocturne C# Min op. Postuma-Frédéric Chopin (3) Prélude n◦ 4—Frédéric Chopin; (4) La mort d’Ase. Peer Gynt-Edvard Grieg; (5) Adagio pour cordes-Samuel Barber; (6) Danse des heures-Amilcare Ponchielli; (7) Le Sacre du printemps-Igor Stravinsky; (8) Dans l’Antre du roi de la montage-Edvard Grieg; (9) Marche Slave-Piotr Ilitch Tchaïkovsky; (10) Danse Arabe-Piotr Ilitch Tchaïkovsky; (11) Danse chinoise-Piotr Ilitch Tchaïkovsky; (12) Le Carnaval des animaux-Camille Saint-Saëns; (13) Au matin-Edvard Grieg; (14) Bolero-Maurice Ravel; (15) Symphonie n 6—Ludwig van Beethoven	The ECS Cycle III spelling test (Evaluation of School Skills, Cycle of Advanced Studies developed by Khomsi, 1998)
Kim & Kim [73]	60	7 to 12	Elementary school	To determine the effects of a musical instrument performance program on emotional intelligence, anxiety, and aggression	Cross-sectional between subjects; experimental (nonequivalent control group pretest-posttest study)	Musical instrument performance program	Emotional intelligence, anxiety, and aggression	Not specified. A children’s song, sonata, or the flute part of an ensemble work for their level.	The Emotional Intelligence Scale, the Trait Anxiety Inventory, and the Aggression Scale
Schellenberg et al. [74]	84	8	Elementary school	To examine whether social benefits are accrued from more typical group music training, specifically an existing program that was designed with music pedagogy as its focus	Cross-sectional between subjects; experimental (controlled trial, non-randomized sample)	Attending public schools that incorporated this specialized program	Emotion comprehension, sympathy and prosocial skills	The repertoire includes arrangements of music taken from classical, jazz, traditional/folk, world, and popular genres	Peabody Picture Vocabulary Test, Test of Emotion Comprehension, and Child-Report Sympathy Scale
Teske et al. [75]	14	8 to 9	Elementary school	To determine the effects of positive mood induction through upbeat music and simulated laughter on creativity	Cross-sectional between subjects; experimental (controlled trial, non-randomized sample)	Listening to a couple of minutes of upbeat instrumental music	Positive mood and humor	Instrumental music with a lively beat	Ad hoc task
Venegas et al. [76]	67	8 to 13	Elementary school	To assess the emotional satisfaction of students while using a cross-disciplinary software that links music and maths	Case study	Use of AudioGraphics software	7 positive and 7 negative (unpaired) emotions	Not specified	Ad hoc instruments
Tricard et al. [77]	83	9 to 10	Elementary school	To examine the influence of inducing positive and negative emotional states on deductive reasoning performances	Cross-sectional between subjects; experimental (controlled trials, non-randomized sample)	To be exposed to video clips and music	Happines and sadness	Music of movies: (1) « Beach Day » of Blanket Barricade; (2) « Adagio for Strings » of Samuel Barber; (3) Background sounds of nature	Facial Action Coding System (Westermann et al., 1996)
Yun & Kim [78]	43	9 to 12	Elementary school	To verify whether the Orff Approach is a proper program to be used with potentially problematic children in order to increase their self-expression, self-efficacy, and social skills	Cross-sectional between subjects; experimental (controlled trial, non-randomized sample)	The Orff Approach method	Self-expression, self-efficacy, self-confidence, self-regulation efficacy and social skills,	Not specified	Self-expression measure (Byun and Kim, 1980); self-efficacy measure (Han, 2002); social skills measure (Mun, 2006)
Katagiri [79]	12	9 to 15	Out of school	To examine the effect of background music and song texts to teach emotional understanding to children with autism	Cross-sectional between groups; experimental (controlled trial, non-randomized sample, counterbalanced treatment)	Background music or singing song, both related to each studied emotion	(1) Type of emotion (happiness, sadness, anger, and fear), and (2) receptive or expressive skill of emotional understanding	Not specified (background music was improvised on the piano and recorded by four pianists skilled at improvisation. Musical cues were taken from Juslin (2000), in which each emotion was associated with a specified tempo, sound level, frequency spectrum, articulation (legato-staccato) and articulation variability. Music recordings representing the four emotions)	Emotional decoding and encoding tasks
Campayo et al. [80]	3	10	Out of school	To examine the relationship between intrapersonal skills and the musical performance of elementary students studying the piano in a Spanish conservatory	Action-research	curricula for the trird year of piano studies	Emotional awareness, regulation and autonomy	Not specified	Ad hoc semi-structured interviews, Teacher´s diary, Video-recordings, CE-360 Assessment protocol
Cook et al. [81]	65	10 to 11	Elementary school	This study evaluated the impact of music-based contact with autistic peers on the attitudes, emotions and behaviors of neurotypical children	Cross-sectional between subjects; experimental (controlled trial, non-randomized sample)	Music-based contact with autistic peers	Prosocial and sympathy	Not specified (They received 11 weekly singing classes − 35 min in length—that were specifically designed to help children develop their social skills, musical engagement and communication between singers)	Social Behaviour Questionnaire (Tremblay et al., 1991) modified; Child-Report Sympathy Scale (Eisenberg et al., 1996) modified; victim scale from the Bullying Prevalence Questionnaire (Rigby and Slee, 1993); bully scale from the Bullying Prevalence Questionnaire (Rigby and Slee, 1993); and ad hoc questionnaire
Lebaka [82]	8	10 to 11	Elementary school	To examine whether selected songs songs are relevant for enriching attitudes and cultural values of children	Descriptive	31 songs collected from Bapedi women. Techniques of playing brass and percussion instruments; singing and reading music; and improvisation	Self-confidence and prosocial	Traditional Pedi religious songs	Ad hoc instruments
Ho et al. [83]	101	10 to 12	Elementary school	To assess the effects of 12 weeks of school counselorled drumming on social-emotional in two fifth-grade intervention classrooms	Cross-sectional between subjects; experimental (controlled trial, non-randomized sample)	Participation in a 12 weeks of school-counselorled drumming program	Emotion management (anxiety, depression, withdrawn, somatic complaints, rule-breaking, etc.)	Not specified (A hybrid of activities used in contemporary drum circles)	Teacher’s Report Form (teacher version of the Child Behaviour Checklist)
Porta [84]	163	10 to 12	Elementary school	To discover the favorite audiovisuals of a sample of 11–12 year old children. To learn what meaning and sense music has for them.	Correlational	Audiovisuals with or without music	Emotional bond	Selection of 14 sequences from the children’s favorite audiovisuals: film; cartoons series; documentary	Ad hoc questionnaires
Kawakami & Katahira [85]	84	11.9	Elementary school	To investigate the emotional mechanisms that make people experience pleasant emotion when listening to sad music	Observational	Listening to two minor pieces considered sad by previous studies; Musical preference	Emotional state (among 50 emotions)	Granados’s Allegro de Concierto (G minor), and Glinka’s La Separation (F minor),	50 emotion-related descriptive words and phrases on a 1 to 5 scale (ad hoc); Interpersonal Reactivity Index for children (Hasegawa et al., 2009)
Su et al. [86]	62	11 to 12	Elementary school	To examine the effects of the Mozart piece K.448 on the learning anxiety, cognitive load, reading rates and reading comprehension of students for reading e-books	Cross-sectional within subjects; experimental (non-randomized sample)	Being exposed to background music (K.448)	Anxiety	Mozart’s piano concerto K.488	Ad hoc reading comprehension tests, leaning anxiety scales from two previous studies (Venkatesh, 2000; He, Chang & Liu, 2010), and a scale of cognitive load (Ouyang et al., 2010)

**Table:** (**B**)

Study	Main Findings	Statistics Significance	Main Limitations
Moore & Hanson-Abromeit [61]	Teachers noted positive change in children’s following the intervention in terms of their emotion skills and peer interactions. Large and medium effects sizes in ER-related abilities were noted, and acceptability and integration of the intervention into the regular daycare environment was supported by interview data	Not applicable	This was a feasibility study, so outcomes should be interpreted with caution due to the small sample size and lack of a comparison group. It should also be noted that this study was conducted with typically developing children at a single daycare site in the southeastern United States, further limiting generalization beyond this study sample. There is no comparison control to demine the efficacy of the MCRF intervention. In the measurement, teachers referenced music in general rather than the MCRF intervention specifically
Boone & Cunningham [62]	Overall, children as young as 4 and 5 years old were able to portray emotional meaning in music through expressive movement. More detailed analysis revealed that this ability was strongest for sad and happy segments and less developed for angry and fearful segments	Significant main effects were found for Age, *F*(1,43) = 4.13, *p* = 0.048, and Emotion, *F*(3,129) = 9.07, *p* < 0.001. Children were more accurate in expressing the emotions of sadness and happiness and less accurate in expressing anger and fear. Additionally, older children were more effective in their communication of emotional meaning.	One potential criticism is that the accurate and identifiable portrayal of a music segment’s emotional meaning through a dance performance does not stipulate that the performer has cognitively “understood” the emotional message. Another possible criticism is that the children could be copying what they saw modeled by the experimenter, rather than encoding the emotional meaning
Brown & Sax [63]	Whithin an arts-integrated program, children showed greater observed positive emotions, and greater growth in teacher-rated levels of positive and negative emotion regulation	Children’s display of positive emotions in arts classes (*M* = 4.35, *SD* = 0.60) versus early learning classes (*M* = 3.95, SD = 1.26) differed significantly (*t*(152) = −3.94, *p* < 0.001). Children’s display of negative emotions in arts (*M* = 0.41, *SD* = 0.51) versus early learning classes (*M* = 0.45, *SD* = 0.86) did not differ significantly (*t*(152) = 0.60, ns)	Results showing an advantage of the arts for children’s emotions and emotion regulation are specific to Settlement Music School’s Kaleidoscope Preschool Arts Enrichment Program, Then the results may not apply to programs that use the arts in other ways. The present research does not uncover how arts exposure might boost emotional functioning, and future research should focus on mechanisms
Habibi et al. [64]	It was found no neural, cognitive, motor, emotional, or social differences among the three groups. In addition, there was no correlation between music perception skills and any of the social or emotional measures	Univariate ANOVA results for each behavioral outcome by group: Mind in the eye: *F*(2,42) = 1.517, *p* = 0.23; Index of empathy: *F*(2,42) = 0.480, *p* = 0.62 Emotion match: *F*(2,42) = 0.502, *p* = 0.60	Gordon’s PMAA is a musical aptitude task that primarily measures perception skills and memory, using an assessment which instead focuses on interpersonal musical experience to correlate with social/emotional skills may be more suitable. It is unclear whether the results are related to cognitive and social and emotional advantages in musicians or the consequence of the participation in an engaging collective activity, or of a combination of various factors.
Ramdane et al. [66]	The majority of the respondents agreed that music and singing help in developing teachers’ thinking skills and raise their emotional awareness	Not applicable	Results limited to Islam education and not generalizable to other educational contexts
Jeremic et al. [67]	Increased social-emotional competencies of students in the experimental group (using the vocal performance teaching method)	Respondents who participated in the instruction carried out by the teacher of music culture showed a statistically significantly higher level of socio-emotional competencies (*p* < 0.05) compared to the control group during the final assessment. At the same time, there were no statistically significant differences in school attendance (Sombor, Kikinda) and gender structure of the respondents.	The limitations of the conducted study was the selection of the sample. Specifically, the results of the study would probably have been more valid if the sample had been random.
Nieminen et al. [65]	School-aged children are able to make emotional and aesthetic judgments about unfamiliar musical pieces	The authors found significant correlations between preference and beauty ratings (r = 0.334, *p* = 0.003), happiness and beauty ratings (r = 0.516, *p* < 0.001), and sadness and ugliness ratings (r = 0.335, *p* = 0.003) for the piece in major (Bonferroni corrected alpha level: *p* = 0.003). A correlation between happiness and beauty rating scores was also found for the minor piece (r = 0.456, *p* < 0.001) and the free tonal one (r = 0.557, *p* < 0.001). Analyzing the groups separately, the authors found significant correlations between happiness and beauty ratings for the major piece (r = 0.475, *p* = 0.003) and the free tonal piece (r = 0.706, *p* < 0.001) in 6–7 year olds. Happiness and beauty ratings were also correlated for the piece in major (r = 0.604, *p* < 0.001) and the piece in minor mode (r = 0.516, *p* < 0.001) in the group of 8–9 year-olds	The music stimuli and musical parameters used in this study were strictly controlled. For this reason this design does not necessarily allow to generalize the results. Moreover, additional refinements regarding the setup and careful modifications of the music material could be used to explore additional developmental aspects of music cognition and appreciation.
Schellenberg & Mankarious [68]	The present findings documented a positive association betweenmusic training and performance on a test of emotional abilities inchildhood. Nevertheless, the link appeared to be a consequence ofhigh levels of cognitive functioning among the musically trainedchildren.	Compared with the untrained children (*M* = 6.86, *SD* = 1.26), the musically trained children (*M* = 7.65, *SD* = 0.81) had higher Test of Emotion Comprehension (TEC) total scores, *t*(58) = 2.86, *p* = 0.006, η^2^ = 0.12.	The vast majority of music training involved private one-on-one lessons, which require solitary practicing between lessons and have no social component other than playing for the instructor. Future research could examine in greater depth the role played by individual versus group lessons in the social-emotional abilities of musically trained children and adults, and whether improved emotional abilities are restricted to emotions conveyed in the auditory domain, or to tests that rely less on cognitive abilities.
Pimenta & Trevisan [69]	An improvement was observed in the feelings experienced when attending these sessions and a new way of relating to the class, greater involvement and interest in it.	Not applicable	The size of the sample. The participating subjects were 31 students from a public school in Sao Paulo, so the results of this study can not be generalized
Rauduvaite [70]	Integrating popular music, active (personal meaning and emotional imitation) methods of music education can be acceptable and efficient for junior school age children	Not applicable	The sample of the educational project included only 70 school learners
Kang [71]	The combination of MI and sandplay was shown to have a positive effect on the improvement of children’s self-expression and emotions	Not applicable	Increase the total number of participants, it would be beneficial to select participants who have a similar developmental level and a proper gender ratio. It may be necessary to have different intervention methods according to the participants’ cognitive developmental levels.
Soulier [72]	Results reveal a negative impact of negative emotional induction on grammatical spelling performances	The effect of the emotional induction factor is significant, (Positive = 33.74%, Negative = 49.58%, Neutral = 39.09%, *F* (2, 216) = 3.01; *p* = 0.05, 2p = 0.03). Post-hoc analysis reveals a significant difference between the percentages of errors obtained under neutral and negative (*p* < 0.03) and negative emotional induction conditions and positive (*p* < 0.001). No interaction is significant.	Limitations regarding emotional induction and measurement
Kim & Kim [73]	The musical instrument performance program improved the ability to perceive emotions, and reduced physical and verbal aggression	There were statistically significant effects of group, *F*(1, 58) = 1.474, *p* = 0.016, time, F(1, 58) = 3.559, *p* = 0.048, and the interaction of group × time, *F*(1, 58) = 4.108, *p* = 0.001, on perceiving emotion (a subcategory of EI), indicating a greater increase in the experimental group compared to the control group during the intervention period. There were statistically significant effects of group, *F*(1, 58) = 1.748, *p* = 0.049, time, *F*(1, 58) = 3.724, *p* = 0.043, and the interaction of group × time, *F*(1, 58) = 4.173, *p* = 0.039, on verbal aggression (a subcategory of aggression), indicating a greater decrease in the experimental group compared to the control group during the intervention period. Physical aggression also showed statistically significant effects of group, *F*(1, 58) = 1.245, *p* = 0.038, and time, *F*(1, 58) = 0.328, *p* = 0.027, but not the interaction effect of group × time, F(1, 58) = 1.129, *p* = 0.176.	All participants were recruited from two elementary schools in Seoul, South Korea using a convenience sampling method. Thus, the participants are not representative of all elementary school children Second, all of the data in this study were from self-reports. Although they guaranteed anonymity and confidentiality to all participants, it is possible that some children might not have answered all the questions honestly. Future studies should include additional informants, such as teachers, peers, and family members.
Schellenberg et al. [74]	Children in the music group had larger increases in sympathy and prosocial , but this effect was limited to children who had poor prosocial skills before the lessons began. The results suggest that groupmusic training facilitates the development of prosocial skills	For emotion comprehension, there was a stronger two-way interaction between testing time and initial performance, *F*(1, 80) = 38.88, *p* < 0.001, partial η^2^ = 0.327. Children who scored low at T1 showed marked improvement from T1 (*M* = 4.41, *SD* = 0.57) to T2 (*M* = 5.52, *SD* = 1.13), *F*(1, 38) = 45.61, *p* < 0.001, partial η2 = 0.546. High scorers showed no change, *p* > 0.2 The results from the ANOVA on sympathy scores revealed a three-way interaction between testing time, initial performance and group assignment, *F*(1, 80) = 4.94, *p* = 0.029, partial η^2^ = 0.058 The analysis of prosocial skills also revealed a three-way interaction between testing time, initial performance and group assignment, *F*(1, 80) = 10.45, *p* = 0.002, partial η^2^ = 0.116	The possibility that an unknown variable caused some of the improvements in social skills that were apparent to the children in the program cannot be ruled out. It is unknown whether non-musical group activities would have a similar effect on prosocial skills. It is also possible that the experimenter’s expectations play a role, with subtle but unintended cues leading children to respond in the desired way. Unfortunately, the music program was implemented in some schools, but not others, making it impossible for experimenters to be blind to group assignment.
Teske et al. [75]	The use of upbeat music and laughing in the experimental condition produced positive mood, thereby facilitating creativity	Results indicated a significant difference with a very large effect size between cartoons made during the experimental condition lessons and cartoons made during the control condition lessons (*p* < 0.001 in humor; *p* = 0.013 in originality). Apparently, lifting students’ moods with upbeat music and laughing affected students’ creative use of humor in their cartoons.	The current study was limited because it considered the broad variable of elevated mood without separating the effects of upbeat music from simulated laughter. The different effects of upbeat and simulated music laughter could be determined by future experiments that examine these variables separately. The small number of children in the sample
Venegas et al. [76]	AudioGraphics generates higher levels of emotional satisfaction than dissatisfaction	There is no significant correlation between positive feelings and appreciation of music and sound (r: 0,201 *p*: 0,104)	They do not present the results on the correlations between negative emotions (Rho Spearman) and musical evaluation
Tricard et al. [77]	It was found a negative effect of sadness on the scores of correct answers to syllogisms compared to joy. Therefore, creating an atmosphere that favors a positive emotion will permit children to better use their cognitive competences	A significant effect of emotional state on performance in deductive reasoning is observed [H (2; *n* = 83) = 14.72; *p* < 0.05]. A significant difference is only observed between the “joy” group and the “sadness” group with [H (2; *n* = 83) = 3.69; *p* < 0.05]. The group “joy” solves on average 5.5 syllogisms correctly (*SD* = 0.82) against 4.25 sylllogisms correctly solved (ET = 1.35) for the group “Sadness”. No other difference is significant	Limitations regarding emotional induction and measurement
Yun & Kim [78]	The results showed that the Orff Approach has a positive effect on children’s self-expression, self-efficacy, and social skills	The Orf Approach was signifcant to the subject’s self-expression (Z = −3.92, *p* < 0.001). Too was significant to the subject’s self-efficacy (Z = −3.93, *p* < 0.001). The efects on the social skills showed a signifcant diference before and after the experiment (Z = −3.89, *p* < 0.001).	The experiment in this research was conducted for 16 sessions, once a week, and looked at the short-term changes and improvements in the children. As the study was targeted at a few low-income children in a limited area it is difficult to generalize
Katagiri [79]	The findings suggest that background music can be an effective tool to increase emotional understanding in children with autism, which is crucial to their social interactions.	The repeated measures analysis revealed that there was a significant difference in understanding among the four emotions (*F* = 4.72, *df* = 3, *p* = 0.01). The repeated measures analysis revealed that there was a significant difference among the intervention conditions (*F* = 9.90, *df* = 3, *p* = 0.00).	The sample size, participants’ diverse ages and verbal abilities, and the time frame for treatment are all factors that may have contributed to the findings of the present study. The sample size was limited to 12 participants so that each counterbalanced treatment order group had only 3 participants.
Campayo et al. [80]	Students who tend to see the positive side of things have higher self-esteem and self-confidence. Teaching with integrated students’ emotional work can improve their performance	Not applicable	This is a case study with three subjects, so its results are not intended to be generalised
Cook et al. [81]	Prosocial emotions towards the social exclusion of a child with autism can be improved for NT children through positive music-based contact. Furthermore, their tendency to be a victim decreases.	Prosocial *z* = −0.14, *p* = 0.89 Sympathy *z* = −0.41 *p* = 0.69 Tendency to be a victim *z* = −0.183 *p* = 0.07 Tendency to be a bully *z* = −0.38 *p* = 0.71	Results from natural experiments are less likely to evidence causation, and while children were broadly matched in terms of socio- economic status (SES), ethnic and cognitive profiles, other unknown variables should not be ruled out. Furthermore, the long-term durability of these findings could not be tested, due to the constraints of this study.
Lebaka [82]	The gains observed include an improved concentration and attitude to schoolwork as well as the unlocking of creative talent	Not applicable	Closer investigation showed that the data on actual composition was difficult to obtain. The origin of these songs is not known. Further investigations should be conducted with regard to the collection, retention and transmission of traditional Pedi religious songs so as not to forever lose these important cultural treasures
Ho et al. [83]	Participation in group drumming led to significant improvements in multiple domains of social-emotional . This sustainable intervention can foster positive youth development and increase student-counselor interaction	Results of the ANOVA Oppositional Defiant Problems (OD). (*F*(3,97) = 3.36, *p* < 0.03; η^2^*p* = 0.09). Post-Traumatic Stress Problems (PT). (*F*(3,97) = 6.40, *p* < 0.01; η^2^*p* = 0.17). Attention Deficit/Hyperactivity Problems (AH). (*F*(3,95) = 5.96, *p* < 0.01; η^2^*p* = 0.14). Anxiety Problems (AN). (*F*(3,96) = 4.97, *p* < 0.01; η^2^*p* = 0.15) Inattention (I—one of two subscales for Attention Deficit/Hyperactivity Problems; the other being Hyper-activity-Impulsivity). (*F*(3,97) = 7.51, *p* < 0.001; η^2^*p* = 0.19).	The effect sizes in the current study (η^2^*p* = 0.09–0.23) were small due to the sample size and inclusionary approach to recruitment; second, random assignment of classrooms to treatment conditions was not feasible due to school administrative constraints; third, teacher raters were not blinded to the group assignment of the students and, thus, may have been prone to reporting bias (this is a major limitation of the pilot study); fourth, the lack of an attention control group and the necessary inclusion of a “gifted” class in the experimental group may have had unintended effects.
Porta [84]	The study shows that sound holds the children’s interest even when the image is removed. Furthermore, music has meaning for children in aspects related to emotions	Not applicable	The sample size. A research line will continue by adding further individual interpretations, in an attempt to extend our knowledge about the communicative, emotional and musical tendencies in childhood and preadolescence.
Kawakami & Katahira [85]	Fantasy was directly associated with liking sad music. Additionally, perspective taking ability, was correlated with the emotional response to sad music. Furthermore, the experience of pleasant emotions contributed to liking sad music.	The authors found that liking sad music was significantly correlated with both Empathic Concern (EC) [*r*(84) = 0.28, *p* < 0.05] and Fantasy (FS) [*r*(84) = 0.27, *p* < 0.05]. In addition, the current data demonstrated that Perspective Taking (PT) was significantly correlated with liking sad music [*r*(84) = 0.35, *p* < 0.01]	First, this study used only sad music as experimental stimuli. It should be carefully assessed whether the relationships between trait empathy and responses to sad music shown in this study were unique to sad music. It is therefore necessary to examine the relationships between the sub-components of trait empathy and music preferences related to emotions other than sadness. Secondly, the developmental aspects of empathic ability might have influenced the results because only elementary school children took part.
Su et al. [86]	This piece of Mozart’s music had positive effect in reducing learning anxiety, and improving the students’ reading rates, reading comprehension and direct process performance	The learning anxiety scores of both groups when Mozart was played were significantly higher than those seen for the silent task, and thus the students’ had lower learning anxiety when listening to this music: Group 1 (*t* = 2.319, *p* < 0.05), Group 2 *t* = 3.148, *p* < 0.01).	This study is based on data obtained using a subjective learning anxiety scale. In order to explore the related issues more objectively, one might consider using an EEG to measure students’ learning anxiety.

**Table 3:** Distribution of identified topics.

Topic		Frequency	Examples
Emotional intelligence	Perception, assessment and expression	42.31%	Recognition, identification, expression, discrimination
	Emotional regulation	7.69%	Management, regulation, reaction
Educational and training benefits		42.31%	Improvements in spelling, mathematics, reading comprehension, motivation, values, creativity
Socio-emotional benefits		26.92%	Reduced Aggressiveness, better empathy, development of pro-social skills
